# Atherosclerotic Calcification Detection: A Comparative Study of Carotid Ultrasound and Cone Beam CT

**DOI:** 10.3390/ijms160819978

**Published:** 2015-08-21

**Authors:** Fisnik Jashari, Pranvera Ibrahimi, Elias Johansson, Jan Ahlqvist, Conny Arnerlöv, Maria Garoff, Eva Levring Jäghagen, Per Wester, Michael Y. Henein

**Affiliations:** 1 Department of Public Health and Clinical Medicine, Umeå University, 90187 Umeå, Sweden; E-Mails: fisnik.jashari@medicin.umu.se (F.J.); elias.johansson@umu.se (E.J.); per.wester@medicin.umu.se (P.W.); michael.henein@medicin.umu.se (M.Y.H.); 2 Department of Pharmacology and Clinical Neuroscience, Umeå University, 90187 Umeå, Sweden; 3 Department of Odontology, Umeå University, 90187 Umeå, Sweden; E-Mails: jan.ahlqvist@umu.se (J.A.); maria.garoff@umu.se (M.G.); eva.levring.jaghagen@umu.se (E.L.J.); 4 Department of Surgical and Perioperative Sciences, Umeå University, 90187 Umeå, Sweden; E-Mail: conny.arnerlov@vll.se

**Keywords:** carotid atherosclerosis, ultrasound, calcification

## Abstract

Background and Aim: Arterial calcification is often detected on ultrasound examination but its diagnostic accuracy is not well validated. The aim of this study was to determine the accuracy of carotid ultrasound B mode findings in detecting atherosclerotic calcification quantified by cone beam computed tomography (CBCT). Methods: We analyzed 94 carotid arteries, from 88 patients (mean age 70 ± 7 years, 33% females), who underwent pre-endarterectomy ultrasound examination. Plaques with high echogenic nodules and posterior shadowing were considered calcified. After surgery, the excised plaques were examined using CBCT, from which the calcification volume (mm^3^) was calculated. In cases with multiple calcifications the largest calcification nodule volume was used to represent the plaque. Carotid artery calcification by the two imaging techniques was compared using conventional correlations. Results: Carotid ultrasound was highly accurate in detecting the presence of calcification; with a sensitivity of 88.2%. Based on the quartile ranges of calcification volumes measured by CBCT we have divided plaque calcification into four groups: <8; 8–35; 36–70 and >70 mm^3^. Calcification volumes ≥8 were accurately detectable by ultrasound with a sensitivity of 96%. Of the 21 plaques with <8 mm^3^ calcification volume; only 13 were detected by ultrasound; resulting in a sensitivity of 62%. There was no difference in the volume of calcification between symptomatic and asymptomatic patients. Conclusion: Carotid ultrasound is highly accurate in detecting the presence of calcified atherosclerotic lesions of volume ≥8 mm^3^; but less accurate in detecting smaller volume calcified plaques. Further development of ultrasound techniques should allow better detection of early arterial calcification.

## 1. Introduction

Carotid atherosclerosis is an important cause of ischaemic stroke [1] and patients with severe stenosis benefit significantly from carotid endarterectomy (CEA) [2]. However, it is well recognised that plaque features add extra diagnostic accuracy for better risk stratification of such patients [3]. According to the conventional atherosclerotic cascade, calcium formation in the carotid wall could be an early pathology that occurs well before significant plaque stenosis and hence is described as subclinical atherosclerosis. Although carotid calcification is detected in 50%–60% of cases [4], often in significantly stenotic lesions (>50%) [5], its association with cerebrovascular events is uncertain. Indeed, controversies exist as to the effect of calcification on plaque nature, with studies suggesting that it both increases stability [6] and is a marker for vulnerability, irrespective of the degree of stenosis [7]. Recently, a systematic review suggested that symptomatic plaques have less calcification compared to asymptomatic plaques [8].

Historically, carotid calcification is detected by plain roentgen imaging. With the development of ultrasound examination of vascular pathology, carotid 2D grey scale has become a routine investigation in vascular laboratories. While ultrasound may detect the presence of calcification, it does not have the accurate means for quantifying its extent. Recently, computed tomography (CT) has become the modality of choice in assessing arterial wall calcification. Even calcification volumes of 1 mm^3^ can be detected and quantified by cone beam CT (CBCT) [9]. However, any CT investigation carries the risk of significant radiation, as well as limited availability. The aim of this study was to determine whether ultrasound (US) B mode could detect carotid calcification quantified by CBCT.

## 2. Results

### 2.1. Clinical Characteristics

Among the 88 patients included in the study, the mean age was 70 ± 7 years and 33% were females. Baseline characteristics of the patients are presented in Table 1. Seventy-three patients had symptomatic carotid stenosis and the remaining 15 patients had asymptomatic carotid stenosis. Six of the symptomatic patients underwent bilateral carotid endarterectomy (CEA); a stenosis severity of 50%–69% was found in six, stenosis severity of 70%–99% in 85 and near-occlusion in three arteries.

**Table 1:** Baseline characteristics of the study population.

Baseline	Study Population (*n* = 88)
Age (years), mean (SD) Females, *n* (%)	70 (7), 29 (33)
Systolic blood pressure (mmHg), mean (SD)	147 (22.6)
Diastolic blood pressure (mmHg), mean (SD)	78 (12)
Total cholesterol (mmol/L), mean (SD)	4.61 (1.03)
LDL (mmol/L), mean (SD)	2.60 (0.92)
HDL (mmol/L), mean (SD)	1.27 (0.48)
Creatinine (μmol/L), mean (SD)	84 (25)
HBA1c (mmol/mol), mean (SD)	52.5 (12.7)
Symptomatic carotid stenosis, *n* (%)	73 (83)
Prior myocardial infarction, *n* (%)	14 (16)
Current angina pectoris, *n* (%)	7 (8)
Heart failure, *n* (%)	1 (1.1)
Previous stroke (>6 months to the present evaluation), *n* (%)	14 (16)
Claudication (lower extremity artery disease), *n* (%)	10 (11.4)
Any previous revascularization for ischemia, *n* (%)	24 (27.3)
Current smoker, *n* (%)	9 (10.2)
Diabetes, *n* (%)	29 (33)
Lipid lowering medicine, *n* (%)	82 (93.2)
Platelet inhibiting or anticoagulation medicine, *n* (%)	88 (100)
Blood pressure reducing medicine, *n* (%)	84 (95.5)

### 2.2. Carotid Calcification: Ultrasound vs. Cone Beam CT (CBCT)

The pre-operative ultrasound examination detected calcification in 82 (87.2%) of the 94 carotid arteries. Calcification volume was quantified with CBCT in 94 CEA specimens and confirmed the presence of calcium in 93 (98.9%). There was no statistically significant difference between the 50%–69% and the 70%–99% degree of stenosis groups (Table 2). Furthermore, there was no difference in carotid calcification between different subgroups according to age, gender or other risk factors (Table 2).

**Figure 1:**
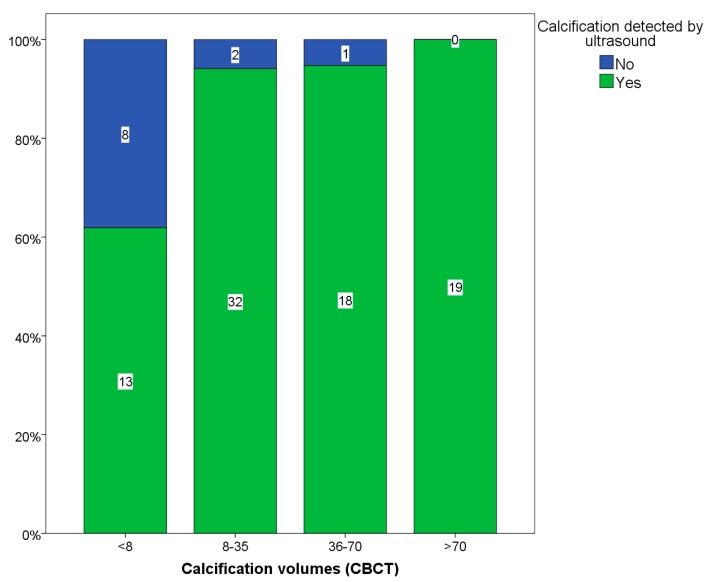
Quartiles of calcification volumes detected and not detected by ultrasound.

Carotid ultrasound was highly accurate in detecting the presence of calcification, with a sensitivity of 88.2%. Using quartiles of calcification volumes measured by CBCT, plaque calcification extent was divided into four groups: <8, 8–35, 36–70 and >70 mm^3^ (Figure 1). Calcification volumes ≥8 mm^3^ were accurately detectable by ultrasound with a sensitivity of 96%. Of the 21 plaques with <8 mm^3^ calcification volume, only 13 were detected by ultrasound, resulting in a sensitivity of 62% (Table 3). There was only one case that was negative for calcification on CBCT but this was also negative on ultrasound (Table 3).

**Table 2:** Subgroup analyses.

Subgroups	Arteries *n*	<8 mm^3^ Calcification Volumes on CBCT, *n* (%)	*p*
Age			
<75	68	18 (26.5)	
≥75	26	4 (15.3)	0.21
Gender			
Female	30	7 (23.3)	
Male	64	15 (23.4)	0.99
Symptomatic	79	20 (25.3)	
Asymptomatic	15	2 (13.3)	0.31
Current smoker			
Yes	10	4 (40.0)	
No	84	18 (21.4)	0.19
Diabetes			
Yes	31	8 (25.8)	
No	63	14 (22.2)	0.60
Previous stroke			
Yes	17	2 (11.8)	
No	77	20 (26.0)	0.21
Previous MI			
Yes	15	3 (20.0)	
No	79	19 (24.0)	0.73
Statin therapy			
Yes	87	21 (24.1)	
No	7	1 (14.3)	0.55
Degree of stenosis			
50%–69%	6	0 (0)	
70%–99%	85	20 (23.5)	
Near-occlusion	3	2 (66.7)	0.08

**Table 3:** Accuracy of Doppler ultrasound in detecting carotid calcification.

CBCT\Ultrasound	Total Arteries, *n*	Calcification on CBCT	Calcification on US	US Sensitivity	US Specificity
Calcification	94	93	82	88.20%	100%
Calcification ≥8 mm^3^	72	72	69	96%	N/A
Calcification <8 mm^3^	22	21	13	62%	100%

N/A: Not applicable.

### 2.3. Carotid Calcification vs. Symptoms

There was no statistical difference in calcification, evaluated by US, between symptomatic and asymptomatic patients. Carotid arteries of symptomatic patients were calcified in 89% of cases compared to 93% in asymptomatic individuals (*p* = 0.51). We then explored whether patients with dispersed calcification within the plaque were more often associated with symptoms compared to those with all the calcification gathered in a solitary nodule, but there was no difference between the groups (*p* = 0.32). There was also no difference in calcification volume quartiles (<8, 8–35, 36–70 and >70 mm^3^) between symptomatic and asymptomatic patients (*p* = 0.35). In addition, we compared calcification volumes <8 mm^3^ on CBCT between different risk factor subgroups but there was no significant difference in volume between those with and without the risk factor (Table 2).

### 2.4. Reproducibility Analyses

The inter-observer agreement for the presence of calcification in the carotid artery stenotic lesions evaluated by US was good (*K* = 0.905, *p* < 0.001).

## 3. Discussion

### 3.1. Findings

Our results show that carotid US is accurate in detecting the presence of calcification in a group of patients with significant stenosis recruited for carotid endarterectomy (CEA). US sensitivity was 96% in identifying calcification volume ≥8 mm^3^ by CBCT. However, calcification volumes <8 mm^3^ were inconsistently detected, with a sensitivity of only 62%. In addition, there was no difference for calcification between symptomatic and asymptomatic patients, neither between other risk factors and degree of stenosis.

### 3.2. Data Interpretation

Calcium formation can be found in any arterial bed. It may be present in the lumen as a calcified plaque cap or may also invade the intima or media [10]. It has been shown that the amount of calcification quantified by carotid CT correlated with respective histology sections [11,12]. Carotid US is an easy and economically favorable method to study different plaque features and their potential association with symptoms [13]. Although it was previously reported that carotid plaque echogenicity, evaluated by US, may not adequately reflect the degree of carotid plaque calcification [14], our study is the first to demonstrate the accuracy of carotid US in identifying calcification deposits and showing that non-detectable calcification is of lower volume and is dispersed throughout the plaque. It was reported that calcification of the carotid plaque is associated with adverse outcomes, such as ipsilateral ischemic stroke, 30 days after carotid artery stenting [15]. Thus, CEA could be feasible in such cases. As many centers now rely mainly on the carotid US findings before intervention, identification of calcification may guide towards better patient risk stratification.

Our findings show that US was accurate in identifying carotid plaques, which after excision proved to have a calcification volume of at least 8 mm^3^. This accuracy was seen in both the two technologies used to assess carotid calcification, CBCT, which directly quantified the calcification volume of extirpated carotid plaques, and US, which qualitatively evaluated plaque echogenicity and posterior shadowing as indirect evidence for plaque calcification. The calcium volume detectable by US (≥8 mm^3^) is relatively significant when compared with normal intima-media thickness, which is usually less than 1 mm, highlighting the accuracy of the US examination in detecting sizable carotid masses. In contrast, the same method failed to detect carotid calcification below a volume of 8 mm^3^ with reliable accuracy. In addition, our findings did not show any statistical difference in the presence of calcification between symptomatic and asymptomatic patients, in keeping with our previous findings [9].

The exact mechanism behind carotid calcification as a potential risk for cerebrovascular events and in predicting strokes still remains controversial [5,6,16]. A systematic review suggested that clinically symptomatic plaques have a lower degree of calcification than asymptomatic plaques [7]. This review had some potential limitations due to the wide range of methods used, highlighting the need for a well-validated, accurate and reproducible technique for calcium quantification. The relationship between arterial calcification and vascular events is more thoroughly studied in the setting of coronary artery disease because of the widely available CT scans performed on patients in cardiology units. Many studies have already shown that the extent of coronary calcification correlates with future development of cardiac events and disease burden [17]. This of course does not refute the presence of a subgroup of patients with calcific coronary artery disease in whom extensive calcification, based on Agatston score classification, is associated with recurrent angina but stable plaques [10,18]. Finally, it has recently been shown that statin therapy increases plaque echogenicity [19] and coronary artery calcification [20]. In our cohort 87.3% of patients were using statin therapy, limiting us in carrying out a reliable comparison between groups.

### 3.3. Clinical Implications

Our findings support the use of US optimum grey scale echogenicity and plaque shadowing as an accurate manifestation of sizable calcification of a minimum volume of 8 mm^3^. US does not appear well suited for the detection of smaller calcium deposits, although they might play a role in plaque vulnerability. Although this method of calcification detection by US is subjective in nature, it establishes a potential foundation for the future development of quantitative models, which could guide towards improved identification of plaque characteristics as a step towards achieving plaque stabilization through optimum treatment. This approach, however, is not sufficiently accurate to detect early calcification pathology based on this biased patient selection. Further development of US plaque studies using methods which quantify echogenicity (grey scale median) could enable detection of earlier stages of calcification and reduce the need for advanced CT examinations and the resultant radiation for patients.

### 3.4. Study Limitations

Because almost all (98.9%) carotid plaques were calcified, as assessed by CBCT, evaluation of US positive and negative predictive value was not possible. We cannot be sure if all calcified material was excised during the CEA procedure but we are working on the assumption that the largest plaque with the largest calcium volume was removed and assessed by CBCT. Furthermore, we cannot be sure that the calcification quantified by CBCT was the same hyperechogenic calcium nodule seen by US in cases with multiple calcifications but we made a further assumption that the nodule with the largest volume was the same nodule as caused the posterior shadowing. In addition, the number of patients with asymptomatic carotid stenosis was small compared with the symptomatic patients, therefore weakening our conclusion in this subgroup. The exact role of stenosis severity in determining accuracy of US in detecting calcification cannot be established since most of our patients had significant (>50%) stenosis.

## 4. Experimental Section

### 4.1. Subjects

This is a secondary analysis of the Doppler US examinations of the Panorama arm of the SPACE study [5]. In the main study, we included 100 patients with significant carotid stenosis (≥50%) who were all eligible for CEA, and who received a pre-operative carotid US examination using the conventional protocol. Patients’ age, gender, recent symptoms and pre-existing co-morbidities, as well as severity of carotid stenosis based on Doppler velocities, were documented. All 100 patients underwent CEA: 94 unilaterally and six bilaterally. Except for five plaques from unilateral CEA, the remainder of the excised plaques was collected, making a total of 101 plaques from 95 patients. This study was approved by the Regional Ethical Board in Umeå (07-004M) and is in compliance with the Helsinki Declaration.

### 4.2. Ultrasound Examination

All patients were examined by experienced ultrasound operators (*n* = 9) and findings were confirmed by a second opinion before reporting. A conventional US system (Acuson Sequoia; Siemens Medical Solutions, Mountain View, CA, USA) equipped with an 8L5 (frequency bandwidth 5–8 MHz) linear-array transducer was used for all carotid examinations. A visible plaque on B-mode with increased Doppler flow velocity was graded according to validated criteria [21], aimed to reproduce North American Symptomatic Carotid Endarterectomy Trial (NASCET) type stenosis for angiography.

### 4.3. Carotid Ultrasound Analysis

**Figure 2:**
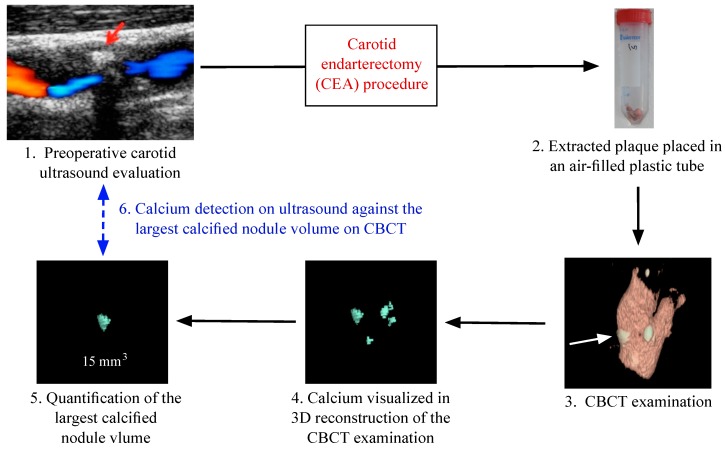
Flowchart of the work process of the study, starting with preoperative ultrasound examination, Cone Beam Computed Tomography (CBCT) evaluation of the carotid specimens removed after carotid endarterectomy (CEA) to images reconstruction and data analysis. Red arrow: hyperechoic spot with posterior shadowing (calcification) on ultrasound. White arrow: calcification on CBCT image.

Two observers blinded to the CBCT results of the excised plaques reviewed all stored US images. A plaque was defined as calcified if there was a hyperechogenic spot with posterior shadowing (Figure 2). Where there was disagreement between the two observers (*n* = 2), the examination was re-evaluated by both observers and consensus was reached.

Seven patients were excluded, five because of suboptimal image quality and two because there was no calcification in the internal carotid artery (ICA) plaque but a distinct calcification in the external carotid artery (ECA). Since it was unclear whether the ECA would be included in the extirpated plaque, it was impossible to make a reasonable assessment without breaking the blinding. The remaining 88 patients constituted the study cohort.

### 4.4. Carotid Endarterectomy (CEA)

All operations were performed under general anesthesia. The common carotid artery and the internal and external carotid arteries were carefully exposed. A longitudinal incision was made in the common carotid artery and this extended into the internal carotid artery and to the healthy artery distal to the plaque. The intimal thickening of the common carotid artery was divided and the plaque was gently dissected from the artery, leaving the adventitial layer intact and taking care to minimize trauma to the plaque. The plaque was further removed from the external carotid artery, along with dissection of the plaque and its calcification until its end in the internal carotid artery. After removal, the plaque was placed in a plastic tube and immediately stored in −20 °C and transferred over to a −80 °C freezer until CBCT analysis.

### 4.5. CBCT

Excised plaques were examined by CBCT (Cone Beam Computed Tomography, 3D Accuitomo 170, J Morita MFG Corporation, Kyoto, Japan; 60 kV, 1 mA, 360°). Due to differences in plaque size, volumes of 4 × 4 or 6 × 6 cm were used and they had a resolution of 0.08–0.125 mm voxel size. The reconstructions were made with 0.5 mm slice thickness and 0.5 mm increment. To counteract partial volume, effect thresholds for maximum window-level were halved in all reconstructions. Volume measurements were performed with a software program (General Electric Company, Barrington, IL, USA, advantage workstation 4.3, Volume Viewer 2) (Figure 1). All calcifications were measured in mm^3^, the resolution of the volume reconstructions were 1 mm^3^. In plaques with multiple calcifications, the largest calcification nodule volume was used to represent the plaque (Figure 2).

### 4.6. Statistics

All statistical analyses were performed using IBM SPSS Statistics 22. Quartile analyses were used for categorizing calcification volume into four groups. Kappa values were calculated to determine the accuracy of US in detecting calcification. Chi-square test was used to compare categorical and ordinal variables with a pre-selected significance level of *p* < 0.05.

## 5. Conclusions

Carotid US is highly accurate in detecting the presence of calcified atherosclerotic lesions of CBCT volume of more than 8 mm^3^. However, it was less accurate in detecting smaller volume calcified plaques, with relatively high false negativity. Calcification was very common in both symptomatic and asymptomatic patients. Further development of US techniques should allow improved detection of early arterial calcification. Since calcification is a dynamic process, finding a method that avoids radiation could be of importance for longitudinal assessment of calcification progression and its effects on plaque stability.
